# Supplementary material for: A Strategy of In Situ Catalysis and Nucleation of Biocompatible Zinc Salts of Amino Acids towards Poly(l-lactide) with Enhanced Crystallization Rate
Source: Polymers (Basel). 2019 May 2;11(5):790. doi: 10.3390/polym11050790 (PMC6572479; doi:10.3390/polym11050790)
Supplement: Supplementary file 1 [file polymers-11-00790-s001.pdf]

Supporting information

A strategy of *in situ* catalysis and nucleation of biocompatible zinc amino acids towards poly(L-lactide) with enhanced crystallization rate

Yuan Liang[1], Meili Sui[2], Maomao He[2], Zhiyong Wei[2,*], and Wanxi Zhang[1]

[1] *School of Materials Science and Engineering, Jilin University, Changchun 130022, China; lyhoneybook@hotmail.com (Y.L.); zhangwanxi0626@sina.com (W.Z.).*

[2] *Department of Polymer Science and Engineering, School of Chemical Engineering, Dalian University of Technology, Dalian 116024, China; 1223918633@qq.com (M.S.); hemao@mail.dlut.edu.cn (M.H.).*

* Correspondence: zywei@dlut.edu.cn; Tel.: +86-411-84986104, ORCID: 0000-0002-5477-9502

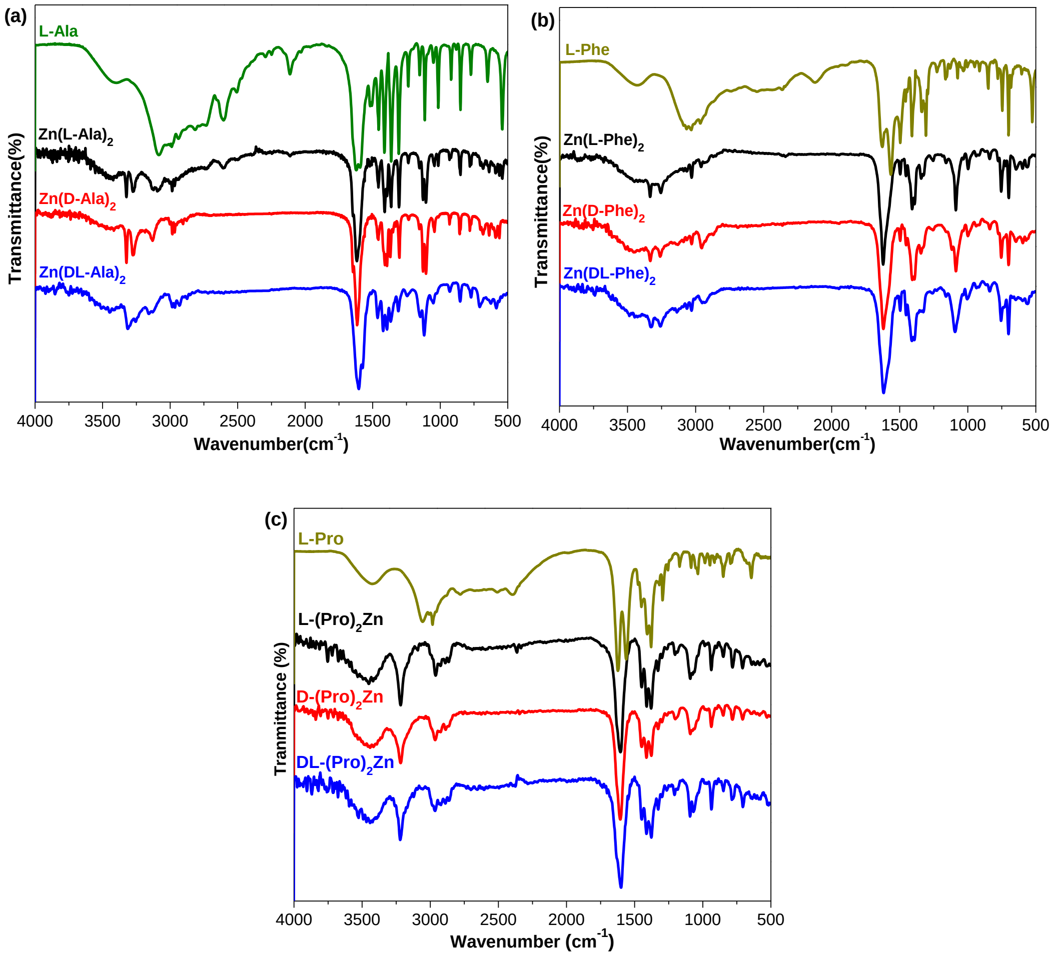

Figure S1 FTIR spectra of (a) Zn(Ala)$_2$, (b) Zn(Phe)$_2$, and (c)Zn(Pro)$_2$

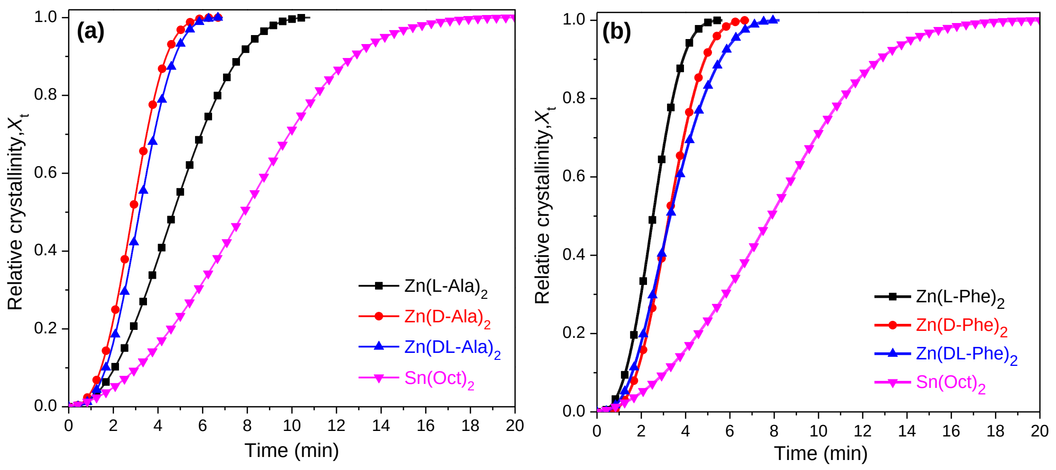

Figure S2 Relative crystallinity as a function of time at 125 °C for PLLA obtained by (a) $Zn(Ala)_2$, (b) $Zn(Phe)_2$, ($Sn(Oct)_2$ as reference).

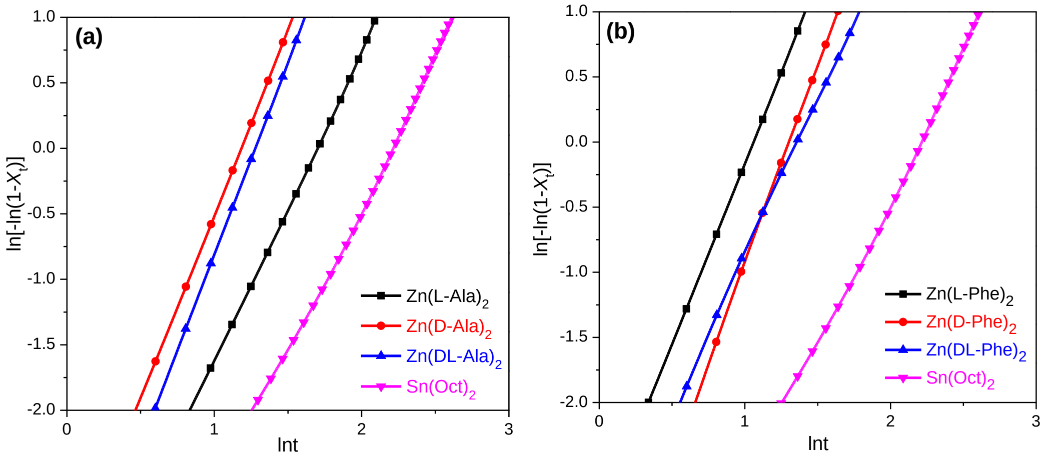

Figure S3 Avrami plots of isothermally crystallized 125 °C for PLLA obtained by (a) $Zn(Ala)_2$, (b) $Zn(Phe)_2$, ($Sn(Oct)_2$ as reference).

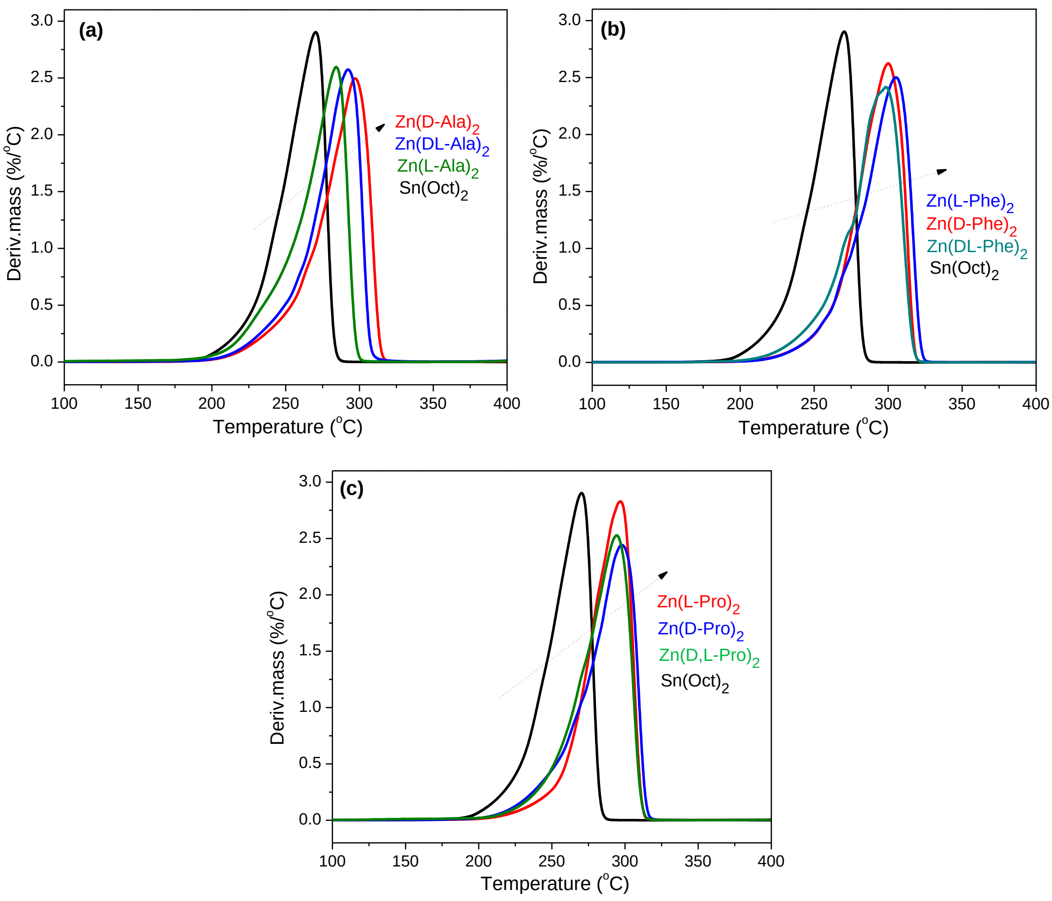

Figure S4 DTG curves of PLLA obtained by (a) $Zn(Ala)_2$, (b) $Zn(Phe)_2$, and (c) $Zn(Pro)_2$
